# Overexpression of a Barley Aquaporin Gene, *HvPIP2;5* Confers Salt and Osmotic Stress Tolerance in Yeast and Plants

**DOI:** 10.3389/fpls.2016.01566

**Published:** 2016-10-21

**Authors:** Hemasundar Alavilli, Jay Prakash Awasthi, Gyana R. Rout, Lingaraj Sahoo, Byeong-ha Lee, Sanjib Kumar Panda

**Affiliations:** ^1^Department of Life Science, Sogang UniversitySeoul, Korea; ^2^Plant Molecular Biotechnology Laboratory, Department of Life Science and Bioinformatics, Assam UniversitySilchar, India; ^3^Department of Agricultural Biotechnology, Orissa University of Agriculture and TechnologyBhubaneswar, India; ^4^Department of Bioscience and Biotechnology, Indian Institute of TechnologyGuwahati, India

**Keywords:** yeast, Arabidopsis, aquaporin, barley, *HvPIP2;5*, overexpression, stress tolerance

## Abstract

We characterized an aquaporin gene *HvPIP2;5* from *Hordeum vulgare* and investigated its physiological roles in heterologous expression systems, yeast and *Arabidopsis*, under high salt and high osmotic stress conditions. In yeast, the expression of *HvPIP2;5* enhanced abiotic stress tolerance under high salt and high osmotic conditions. Arabidopsis plants overexpressing *HvPIP2;5* also showed better stress tolerance in germination and root growth under high salt and high osmotic stresses than the wild type (WT). *HvPIP2;5* overexpressing plants were able to survive and recover after a 3-week drought period unlike the control plants which wilted and died during stress treatment. Indeed, overexpression of *HvPIP2;5* caused higher retention of chlorophylls and water under salt and osmotic stresses than did control. We also observed lower accumulation of reactive oxygen species (ROS) and malondialdehyde (MDA), an end-product of lipid peroxidation in *HvPIP2;5* overexpressing plants than in WT. These results suggest that *HvPIP2;5* overexpression brought about stress tolerance, at least in part, by reducing the secondary oxidative stress caused by salt and osmotic stresses. Consistent with these stress tolerant phenotypes, *HvPIP2;5* overexpressing Arabidopsis lines showed higher expression and activities of ROS scavenging enzymes such as catalase (CAT), superoxide dismutase (SOD), glutathione reductase (GR), and ascorbate peroxidase (APX) under salt and osmotic stresses than did WT. In addition, the proline biosynthesis genes, Δ^*1*^*-Pyrroline-5-Carboxylate Synthase 1* and *2* (*P5CS1* and *P5CS2*) were up-regulated in *HvPIP2;5* overexpressing plants under salt and osmotic stresses, which coincided with increased levels of the osmoprotectant proline. Together, these results suggested that *HvPIP2;5* overexpression enhanced stress tolerance to high salt and high osmotic stresses by increasing activities and/or expression of ROS scavenging enzymes and osmoprotectant biosynthetic genes.

## Introduction

Aquaporins belong to major intrinsic proteins (MIPs) that are present from prokaryotes to plants and animals. These proteins facilitate the transport of water and small uncharged molecules across biological membranes (Park and Saier, 1996; Heymann and Engel, 1999; Engel and Stahlberg, 2002; Zardoya et al., 2002; Maurel et al., 2008, 2015). MIPs are categorized into two groups: aquaporins that show water-specific channel activity and glycerol-uptake facilitators (GLPs or aquaglyceroporins) that have channel activity for additional small molecules such as glycerol or urea (Park and Saier, 1996; Heymann and Engel, 1999; Engel and Stahlberg, 2002; Zardoya et al., 2002; Zardoya, 2005; Maurel et al., 2008). In plants, all MIPs, except for GlpF-like intrinsic proteins (GIPs), exhibit water-specific channel activity; therefore, most plant MIPs are collectively called aquaporins (Maurel et al., 2008, 2015). Recently, it has been shown that plant MIPs can transport other small molecules such as CO_2_ and ammonia (Uehlein et al., 2003; Jahn et al., 2004).

Compared to genomes of other organisms, plant genomes contain a higher number of aquaporins (Wang et al., 2014). For instance, there are 35 aquaporin genes in *Arabidopsis thaliana* (Maurel, 2007), 22 in *Jatropha curcas* (Khan et al., 2015), 36 in *Zea maize* (Chaumont et al., 2001) and over 40 in *Hordeum vulgare* (Hove et al., 2015) while *Escherichia coli* (Gomes et al., 2009), *Caenorhabditis elegans* (Ishibashi et al., 2011), *Drosophila melanogaster* (Spring et al., 2009), and *Homo sapiens* (Day et al., 2014) contain 2, 11, 7, and 12, respectively.

The high diversity in plant aquaporins suggests variation of their physiological roles. Indeed, aquaporins were shown to be associated with vital physiological processes such as photosynthesis, nitrogen fixation, nutrient uptake and other environmental stress responses (Li et al., 2014; Hove et al., 2015; Sun et al., 2015). Plant aquaporins are classified into five subgroups, i.e.,: the plasma membrane intrinsic proteins (PIPs), tonoplast intrinsic proteins (TIPs), nodulin26-like intrinsic proteins (NIPs), small basic intrinsic proteins (SIPs) and the recently identified uncategorized (X) intrinsic proteins (XIP) (Maurel et al., 2015). Based on sequence divergence, PIPs are further divided into PIP1 and PIP2 subclasses each consisting of several isoforms which play important roles in determining hydraulic conductivity particularly in roots (Martre et al., 2002; Siefritz et al., 2002; Javot et al., 2003; Postaire et al., 2010). Analyses on PIP1 and PIP2 from barley and maize revealed that the PIP2 proteins had higher water transport activity than PIP1 proteins in *Xenopus* oocytes (Chaumont et al., 2000; Horie et al., 2011). When PIP2 was co-expressed with functional or even nonfunctional PIP1 proteins, water transport activity of PIP2 was enhanced (Chaumont et al., 2000; Fetter et al., 2004; Horie et al., 2011). This enhanced water transport was attributed to their ability to form heterotetramers for proper trafficking to the plasma membrane (Fetter et al., 2004; Zelazny et al., 2007).

Dynamic changes in the expression levels of many *PIP* genes were observed in response to drought stress, suggesting their involvement in stress responses by regulating water balance (Afzal et al., 2016). Studies with *PIP*-defective mutants or overexpressing plants also linked the roles of PIP proteins to water-deficit stress tolerance. For example, when expression of a tobacco *PIP1* member (*NtAQP1*) was reduced by antisense technology, the *NtAQP1*-downregulated tobacco plants showed reduced root hydraulic conductivity and wilting phenotypes under water stress (Siefritz et al., 2002). *Physcomitrella patens PIP2;1* or *PIP2;2* knockouts showed low water permeability with drought-sensitive phenotypes (Lienard et al., 2008). Reduction in water permeability of protoplasts and root hydraulic conductivity were observed respectively in Arabidopsis *PIP1;2* and *PIP2;2*-defective mutants but without clear developmental defects (Kaldenhoff et al., 1998; Javot et al., 2003). Overexpression of several *PIP* genes from various plants including *Oryza sativa, Vicia faba, Nicotiana tabacum*, and *Triticum aestivum* successfully enhanced water stress tolerance in transgenic plants (Lian et al., 2004; Cui et al., 2008; Sade et al., 2010; Zhou et al., 2012). Interestingly, some contrasting results (i.e., stress sensitive phenotypes in *PIP* overexpressing plants) have also been reported, implying the complexity of *PIP* function in plants (Aharon et al., 2003; Katsuhara et al., 2003; Jang et al., 2007; Li et al., 2015).

Barley (*Hordeum vulgare* L.) is one of the most agronomically cultivated crops; it is more adaptable to drought, salinity and cold than other cereal crops (Katsuhara et al., 2014; Hove et al., 2015). These characteristics would possibly make the barley gene pool, including barley aquaporins, as one of stress-adaptive genetic resources. Although, several *PIPs* have been identified in barley, only few of them have been functionally characterized thus far.

In this study, we overexpressed barley *PIP2;5* (*HvPIP2;5*) in yeast and Arabidopsis and characterized these lines to understand the functions of the barley *PIP* gene under high salt and high osmotic stress conditions.

## Materials and methods

### *HvPIP2;5* expression vector construction

Barley (*Hordeum vulgare* cv. NP21) cDNA was prepared using superscriptTM III reverse transcriptase (Invitrogen, USA), and total RNA was extracted with TRIzol® Reagent (Ambion, USA). A 873 bp-length *HvPIP2;5* coding sequence (GenBank Accession number: AB377270.1) was cloned into TA cloning vector pTOPO2.1 (Invitrogen, Carlsbad, CA, USA) using gene specific primers (Supplementary Table 1). The coding sequences of *HvPIP2;5* was cloned into yeast expression vector pYES2.0 (Invitrogen, USA) at the EcoRI site and named pYES2: *HvPIP2;5.* For plant transformation, *HvPIP2;5* coding sequence was cloned into a standard plant binary vector pCAMBIA2301. The resulting overexpression construct was named pCAMBIA2301-35S:*HvPIP2;5.*

### Transformation of yeast and stress analysis

The *HvPIP2;5* coding sequence under control of GAL1 promoter in pYES2 yeast expression vector was introduced into yeast FY3 cells. As controls, FY3 cells containing pYES2 vector only (vector control) and FY3 strain only were used in stress assays. The yeast strain FY3 was transformed with a pYES2 empty vector or pYES2:*HvPIP2;5* recombinant vector by lithium acetate method (Kawai et al., 2010) and selected on SC medium devoid of uracil. Yeast cells expressing *HvPIP2;5* along with control cells were grown on YPD solid medium (1% Yeast extract, 2% peptone, and 2% glucose).

For stress analysis, transformed yeast cells were propagated in SC-U medium containing 2% galactose for 12 h and cell density was adjusted to 1.0 of OD600 followed by serial dilutions. Yeast cell were spotted on YPD medium supplemented with PEG (4%) or NaCl (200 mM), respectively. Plates were maintained at 30°C and growth was monitored after 2 days.

### Generation of *HvPIP2;5* overexpressing arabidopsis lines and stress analysis

Using *Agrobacterium tumefaciens* GV3101 harboring pCAMBIA2301-35S:*HvPIP2;5*, Arabidopsis Columbia-0 plants were transformed via floral dipping method (Clough and Bent, 1998). Homozygote for 35S:*HvPIP2;5* insertion was selected at T4 generation by analyzing kanamycin resistance at each generation.

For germination analysis, the seeds of WT and homozygote *HvPIP2;5* OE Arabidopsis lines were plated on half strength Murashige Skoog (MS) media and allowed to germinate at 22°C and 60% relative humidity with a 16/8 h light-dark photo cycle after 3 day stratification at 4°C.

Final concentration of 100–200 mM NaCl (for high salt stress) or 10–20% PEG (for high osmotic stress) was supplemented to the MS media for stress administration. Hereby salt stress or osmotic stress indicates high salt stress or high osmotic stress, respectively. Emergence of cotyledons was used as a germination criterium. The number of germinated seeds was expressed as a percentage of total number of seeds planted after 7 days. For root elongation analysis, vertically grown seedlings with 1–1.5 cm long root were transferred onto a MS vertical plate supplemented with or without stress agents (200 mM NaCl and 20% PEG). Root length was measured 15 days after transfer. Root length of seedlings under stress conditions was expressed as a percentage of their respective controls grown on normal MS medium. All stress analysis experiments were conducted three times and each contained 3 biological repeats. For drought test with soil-grown plants, 4 week old seedlings were subjected to drought stress by withholding water supply for 21 days and then re-watered. For salt and osmotic stress, 3 week old plants were irrigated with either half strength MS liquid (control) or half strength MS liquid supplemented either with 200 mM NaCl or 20% PEG to impose stress for 15 days at 2 day intervals and then harvested for analysis. The chlorophyll, proline, and malondialdehyde (MDA) content in control or stress treated plants were determined by the methods reported previously by Lichtenthaler (1987), Bates et al. (1973), and Heath and Packer (1968), respectively.

### Water loss and relative water content analysis

Rosette leaves of 3 week old seedlings were detached, weighed and placed on paper under the fume hood to administrate drought stress. Fresh weights of rosette leaves were measured each hour for 5 h. Water loss was calculated as the loss in fresh weight of the samples. For relative water content analysis, the root from WT and the *HvPIP2;5* OE lines were excised and treated either with 200 mM NaCl or 20% PEG or deionized water after measuring the fresh weight. After 24 h of incubation, the tissues were weighed again for their turgid weight and then dried completely to obtain dry weight. The root RWC was calculated by the following formula; RWC (%) = [(fresh weight − dry weight)/(turgid weight − dry weight)] × 100 (Weatherley, 1950).

### Reactive oxygen species analysis and antioxidant enzyme activity assay

*In situ* detection of superoxide by nitro blue tetrazolium (NBT) staining and hydrogen peroxide by 3, 3'diaminobenzidine (DAB) staining was performed according to methods previously described by Rao and Davis (1999) and Ramel et al. (2009). For this, 3 week old seedlings were grown on MS medium with 0, 100, 200 mM NaCl and the third leaf from the top was used. Superoxide radicals were visualized as blue color produced by NBT precipitation while hydrogen peroxide spots were visualized as brown color due to DAB polymerization.

For ROS quantification and antioxidant enzyme activity assay, 3 week old plants were irrigated with either half strength MS liquid (control) or half strength MS liquid supplemented either with 200 mM NaCl or 20% PEG to impose stress for 15 days at 2 day intervals and then harvested for analysis. Contents of superoxide and hydrogen peroxide were estimated by methods previously described by Elstner and Heupel (1976) and Sagisaka (1976), respectively. For antioxidant enzyme assay, the samples (200 mg tissue) were homogenized in 1.5 mL of 0.1 M phosphate buffer (pH 6.8) containing 1 mM ethylenediamine tetra-acetic acid (EDTA) and 1% polyvinylpyrrolidone (PVP) in a chilled pestle and mortar. The homogenate was then centrifuged at 17,000 g for 15 min at 4°C. The supernatant was quantified by the Bradford method (Bradford, 1976) and used for the assay of catalase (CAT), superoxide dismutase (SOD) and glutathione reductase (GR) (Chance and Maehly, 1955; Smith et al., 1988; Gupta et al., 1993). For the ascorbate peroxidase (APX) activity assay, a final concentration of 1 mM ascorbic acid was added to the assay buffer (Nakano and Asada, 1981).

### Gene expression analysis

Plants were treated with water for control or 300 mM NaCl for 6 h or 20% PEG for 6 h, and total RNAs were extracted using RNeasy Plant mini Kit (Qiagen, Germany). cDNA was synthesized using RevertAid First Strand cDNA Synthesis Kit (ThermoFisher, USA), and resulting cDNAs were used for semi-quantitative RT or qRT-PCR experiments for selected genes. Primer pairs used in experiments are shown in Supplementary Table 1.

### Statistical analysis

All statistical comparisons between variances were determined by ANOVA (Analysis of variance) and least significant differences (LSD) between variants were calculated using Statistix 8.1 computation software. Statistically significant mean values were denoted as * (*P* ≤ 0.05).

## Results

### Sequence analysis of *HvPIP2;5*

Previously, we identified several barley plasma membrane intrinsic protein (*HvPIP*) genes and examined their expressions under salt and osmotic stress conditions (Horie et al., 2011; Katsuhara et al., 2014). For further functional and physiological characterization, we selected *HvPIP2;5* (Genbank #AB377270), one of the *HvPIP2* genes that possesses abundant transcripts and demonstrates down-regulation of gene expression under salt and osmotic stresses (Horie et al., 2011; Katsuhara et al., 2014). The *HvPIP2;5* gene encodes a polypeptide of 291 amino acids with an estimated molecular mass of 30.3 KDa and an isoelectric point of 8.28 as predicted by ExPaSy bioinformatics tools for protein structure analysis (http://web.expasy.org/compute_pi/). HvPIP2;5 protein shares 81% identity with Arabidopsis PIP2;5 protein (AtPIP2;5, AT3G54820). As shown in Supplementary Figure 1A, both HvPIP2;5 and AtPIP2;5 harbor two Asn-Pro-Ala (NPA) motifs in addition to the highly conserved amino acid sequence “HINPAVTFG” which is reportedly conserved among all the MIP superfamily proteins (Li et al., 2009; Zhou et al., 2012). Conserved phosphorylation-target serine residues were also found in the C-termini of both *PIP2;5* orthologs (Supplementary Figure 1A; Hove et al., 2015). The predicted HvPIP2;5 protein contained aquaporin-characteristic six transmembrane spanning α-helices (H1-H6) with presence of 35.17% alpha helix, 30% random coil, 24.48% random coil, and 10.34% beta turn (Supplementary Figures 1B,C).

It has been recently reported that the barley genome comprises at least 40 aquaporin genes with 5 PIP1 genes and 9 PIP2 genes (Hove et al., 2015). Multiple sequence alignment using all reported HvPIP2 isoforms was performed using Mega 6 software (http://www.megasoftware.net). As expected, HvPIP2 proteins showed high degree of homology among them, and HvPIP2;5 was located in the same clade as HvPIP2;1 with 85% identity to HvPIP2;1 (Supplementary Figure 1D).

### Increased tolerance of *HvPIP2;5*-expressing yeast under high salt and osmotic stresses

**Figure 1:**
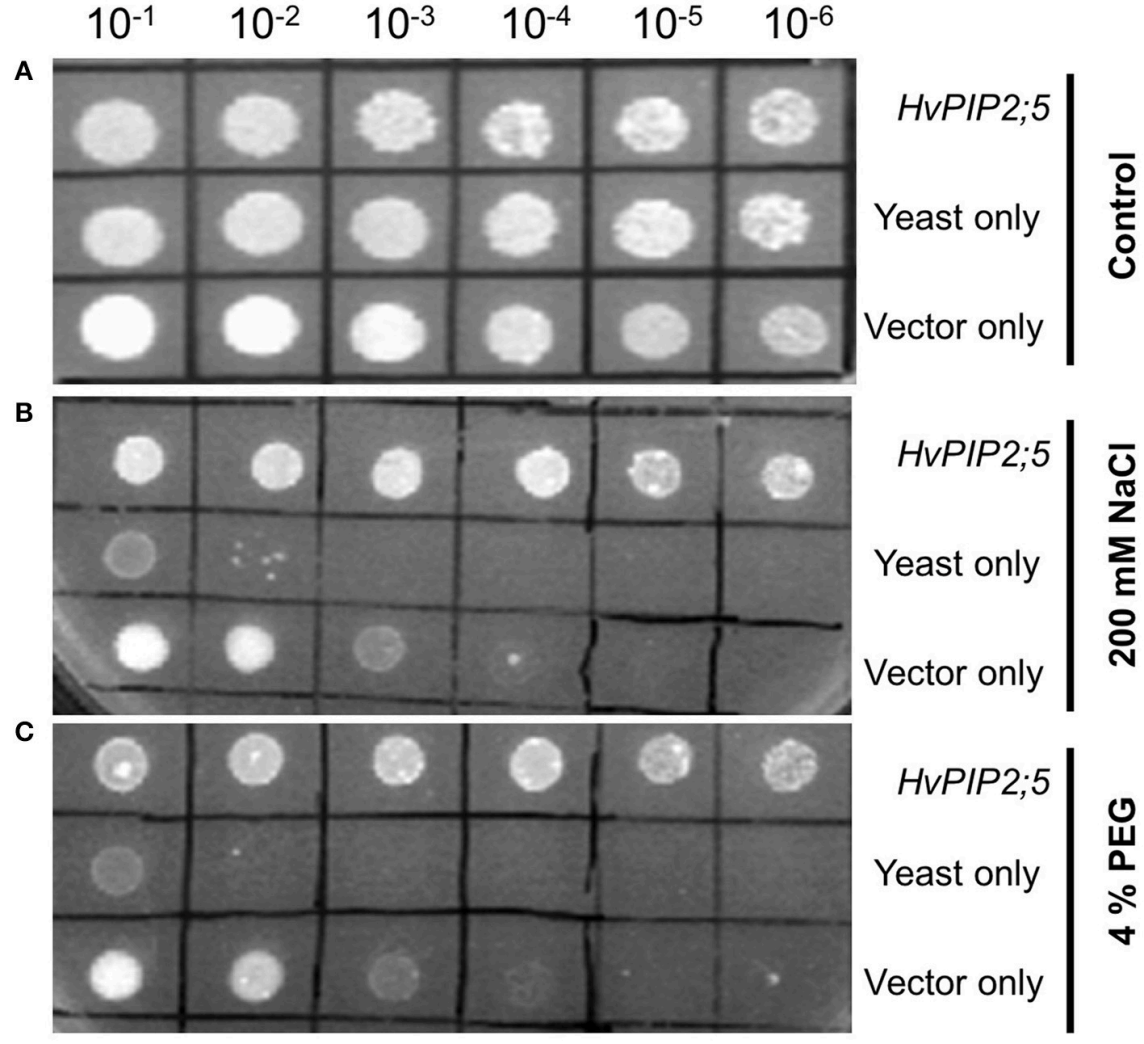
**Effect of HvPIP2;5 expression in yeast under salt and osmotic stresses**. Yeast cells harboring the *HvPIP2;5* expressing construct (HvPIP2;5), yeast cells only (yeast), and yeast cells with the vector pYES2 only (vector only) were subjected to 200 mM NaCl and 4% PEG. Cell density was adjusted to OD-600 at 1.0 and serial dilutions were made at each step. Ten microliter of each dilution was spotted on **(A)** YPD plates without stress. **(B)** YPD plates supplemented with 200 mM NaCl. **(C)** Supplemented with 4% PEG. Photographs were taken after 48 h of incubation at 30°C.

A yeast expression system was employed to examine the functions of the *HvPIP2;5* gene under high saline and high osmotic conditions. As shown in Figure 1A, yeast strains transformed with *HvPIP2;5* or empty vector and FY3 cells could grow up to 10^−6^ dilution on YPD plates. In the presence of 200 mM NaCl, pYES2 vector-containing FY3 cells grew only until 10^−3^ dilution, whereas the yeast strain transformed with *HvPIP2;5* was able to grow until 10^−6^ dilution (Figure 1B). Similarly, empty vector containing cells grew only to 10^−3^ dilution on the YPD medium supplemented with 4% polyethylene glycol (PEG), while yeast cells with *HvPIP2;5* displayed growth until 10^−6^ dilution under the same condition (Figure 1C). Taken together, these results showed that *HvPIP2;5* expression in yeast resulted in increased stress tolerance under high salt and high osmotic conditions.

### Enhanced stress tolerance of *HvPIP2;5*-overexpressing arabidopsis

To investigate the functional roles of *HvPIP2;5 in planta*, we expressed *HvPIP2;5* in Arabidopsis under the control of CaMV 35S promoter (Supplementary Figure 2A). Two independent homozygous *HvPIP2;5* overexpressing (OE) lines were selected. PCR analysis confirmed the presence of the *HvPIP2;5* gene in transgenic plants (data not shown) and semi quantitative RT-PCR for *HvPIP2;5* expression analysis demonstrated that transgenic lines were overexpressing *HvPIP2;5* gene (Supplementary Figure 2B).

**Figure 2:**
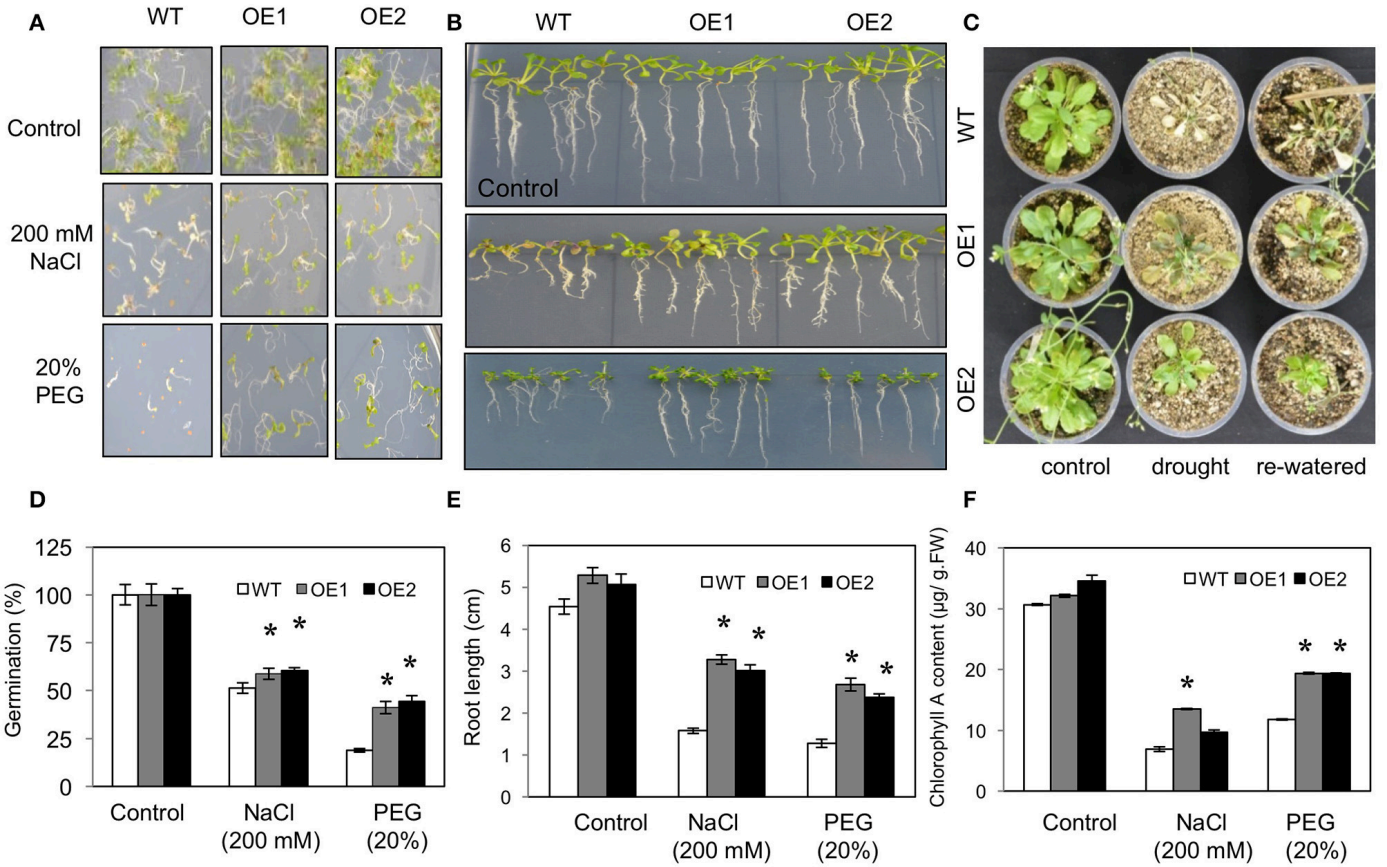
**Phenotypes of *HvPIP2;5* overexpressing Arabidopsis under salt and water-deficit stresses**. WT and two *HvPIP2;5* overexpressing Arabidopsis lines (OE1,OE2) were used for analysis. **(A,D)** Seed germination of WT and OE lines on MS media containing 200 mM NaCl or 20% PEG. The number of germinated seeds was expressed as a percentage of total number of seeds planted. **(B,E)** Root elongation on WT and OE lines on MS media containing 200 mM NaCl or 20% PEG. Pictures for root elongation comparison were taken 21 days after planting. **(C)** Effects of drought on WT and OE lines. Four week old seedlings were subjected to drought stress by withholding water supply for 21 days and then re-watered. **(F)** Measurement of chlorophyll A in WT and OE lines after 200 mM NaCl or 20% PEG treatment. Bars indicate standard error and significant differences between WT and OE lines were marked with asterisks (*P* < 0.05).

Germination under 200 mM NaCl (high salt stress) and 20% PEG (high osmotic stress) was first tested for two independent *HvPIP2;5* OE lines along with WT. On the control medium, all lines germinated successfully after 7 days of planting. On the medium supplemented with 200 mM NaCl, WT germination ratios were decreased to about 50%, whereas *HvPIP2;5* OE lines displayed a 59–60% germination ratio on salt medium (Figures 2A,D). Also, *HvPIP2;5* OE lines displayed a 41–44% germination ratio in MS medium with 20% PEG whereas WT germination was reduced to 18% (Figures 2A,D).

Root growth under salt and osmotic stress conditions was also compared. In root growth assays, we observed increased tolerance of *HvPIP2;5* OE lines under salinity and osmotic stresses compared to WT. The relative root length of *HvPIP2;5* OE lines was significantly higher in the presence of 200 mM NaCl (59–62% vs. 34%) or 20% PEG (47–50% vs. 28%) compared to that of WT (Figures 2B,E).

We further evaluated growth performance of WT and *HvPIP2;5* OE lines during drought stress. WT and *HvPIP2;5* OE plants were grown in well-watered conditions for 4 weeks and then subjected to drought conditions. After 21 days of water withdrawal, WT lines became wilted with retarded growth, whereas *HvPIP2;5* OE lines did not wilt as severely as WT. Upon re-watering, WT plants were so severely damaged that they were unable to resume growth. In contrast, the transgenic lines were able to recover and retained survival upon rehydration (Figure 2C).

Chlorophyll degradation is among the changes caused by salt and osmotic stresses. No remarkable differences were observed between WT and *HvPIP2;5* OE lines grown under control conditions; however, after stress treatments with 200 mM NaCl or 20% PEG, *HvPIP2;5* OE lines showed less chlorophyll degradation when compared with WT. Measurement of chlorophyll contents of stress-treated plants confirmed the better retention of chlorophyll A in OE lines than in WT (Figure 2F). WT under salinity stress with 200 mM NaCl contained only 23% of chlorophyll A levels under normal conditions, while OE lines under salt stress had largely higher levels of chlorophyll A retention (27–43%). Additionally, osmotic stress with 20% PEG caused 51 and 25–31% chlorophyll A loss in WT and *HvPIP2;5* OE, respectively (Figure 2F).

### Low water loss and high water retention in *HvPIP2;5* overexpressing lines under stresses

**Figure 3:**
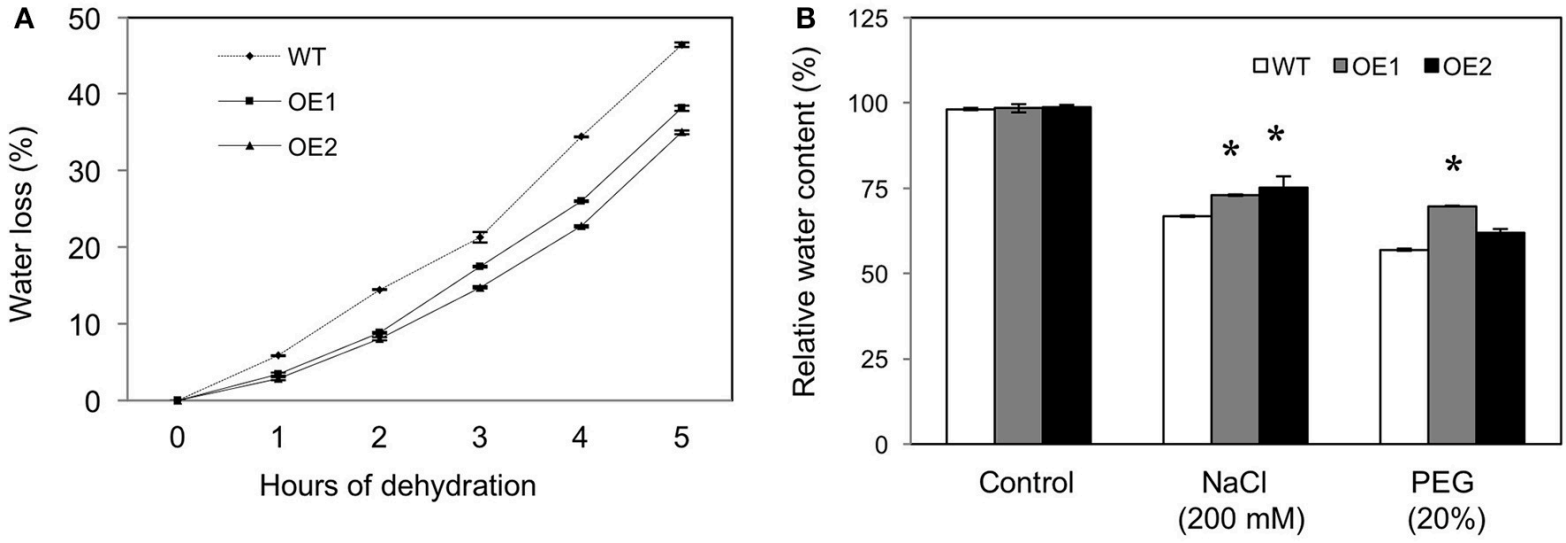
**Water loss and retention in *HvPIP2;5* overexpressing Arabidopsis under water stress. (A)** Comparison of water loss from detached rosette leaves of WT and OE lines, control plants. Water loss was calculated from the loss in fresh weight of the samples. **(B)** Relative water content estimation in roots of WT and OE lines after 200 mM NaCl or 20% PEG treatment. Bars indicate standard error and significant differences between WT and OE lines were marked with asterisks (*P* < 0.05).

Dehydration tolerance in *HvPIP2;5* lines was assessed by measuring the water loss percentage. For this, rosette leaves from WT and *HvPIP2;5* OE lines were detached, placed on paper for dehydration and fresh weight measured. After 5 h of dehydration, the leaves of WT plants lost ~45% of their original fresh weight, whereas leaves of the *HvPIP2;5* OE lines lost only about 30–35% from their initial fresh weight (Figure 3A).

In addition, relative water content (RWC) was measured from the root tissues of WT and *HvPIP2;5* OE lines under salt or osmotic stress. Under salt stress with 200 mM NaCl, WT lines held 68.1% RWC, whereas *HvPIP2;5* OE lines kept 75–77% RWC (Figure 3B). Likewise, OE lines contained largely higher RWC than did WT under osmotic stress with 20% PEG (Figure 3B).

### Reduction of oxidative stress in *HvPIP2;5* overexpressing lines under stresses

**Figure 4:**
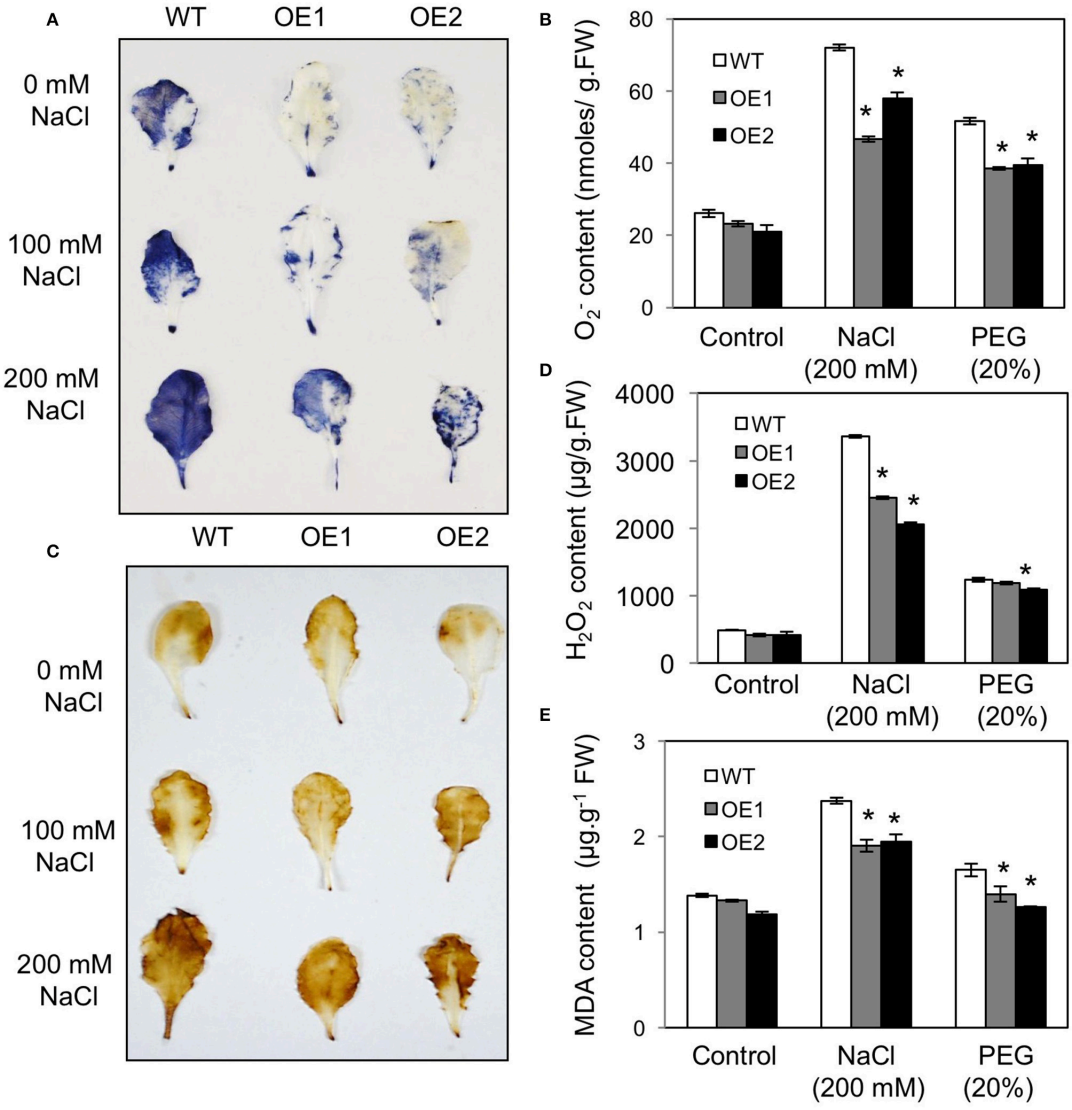
**Analysis of superoxide, hydrogen peroxide, malondialdehyde contents in *HvPIP2;5* overexpressing lines under salt and osmotic stresses. (A)** NBT (nitro blue tetrazolium) staining for superoxide detection in WT and OE lines after NaCl treatment. **(B)** Quantification of superoxide contents in WT and OE lines after 200 mM NaCl or 20% PEG treatment. **(C)** DAB (3, 3-diaminobenzidine) staining for hydrogen peroxide detection in WT and OE lines after NaCl treatment. **(D)** Quantification of hydrogen peroxide contents in WT and OE lines after 200 mM NaCl or 20% PEG treatment. **(E)** Quantification of MDA (malondialdehyde) levels in WT and OE lines after 200 mM NaCl or 20% PEG treatment. Bars indicate standard error and significant differences between WT and OE lines were marked with asterisks (*P* < 0.05).

Reactive oxygen species (ROS) are generated in plants under drought, salt, and temperature stresses. Using nitro blue tetrazolium (NBT) staining for superoxide and diaminobenzidine (DAB) staining for hydrogen peroxide, we measured the levels of ROS in WT and *HvPIP2;5* OE lines under high salt (200 mM NaCl) and high osmotic stress (20% PEG). Compared to WT, the *HvPIP2;5* OE lines showed significantly weaker NBT staining and less ${\text{O}}_{2}^{-}$ amount under osmotic or salt stress conditions (Figures 4A,B). Similarly, DAB staining and quantification of H_2_O_2_ content revealed lower amounts of H_2_O_2_ in the OE lines than in WT after NaCl treatment (Figures 4C,D). However, *HvPIP2;5* OE lines did not seem to display very significant reduction in H_2_O_2_ levels after PEG treatment when compared to WT.

Malondialdehyde (MDA) is an end product of lipid peroxidation of cell membrane lipids and a good indicator of oxidative damage (Diao et al., 2011). After salt stress (200 mM NaCl) and osmotic stress (20% PEG), MDA content was significantly lower in *HvPIP2;5* OE lines than in WT, which implicates lesser membrane damage in *HvPIP2;5* OE lines (Figure 4E). These results suggested that enhanced stress tolerance in *HvPIP2;5* OE lines might be due, at least in part, to reduced levels of oxidative stress caused by salt and osmotic stresses.

### Increased activities of ROS scavenging enzymes in *HvPIP2;5* overexpressing lines

**Figure 5:**
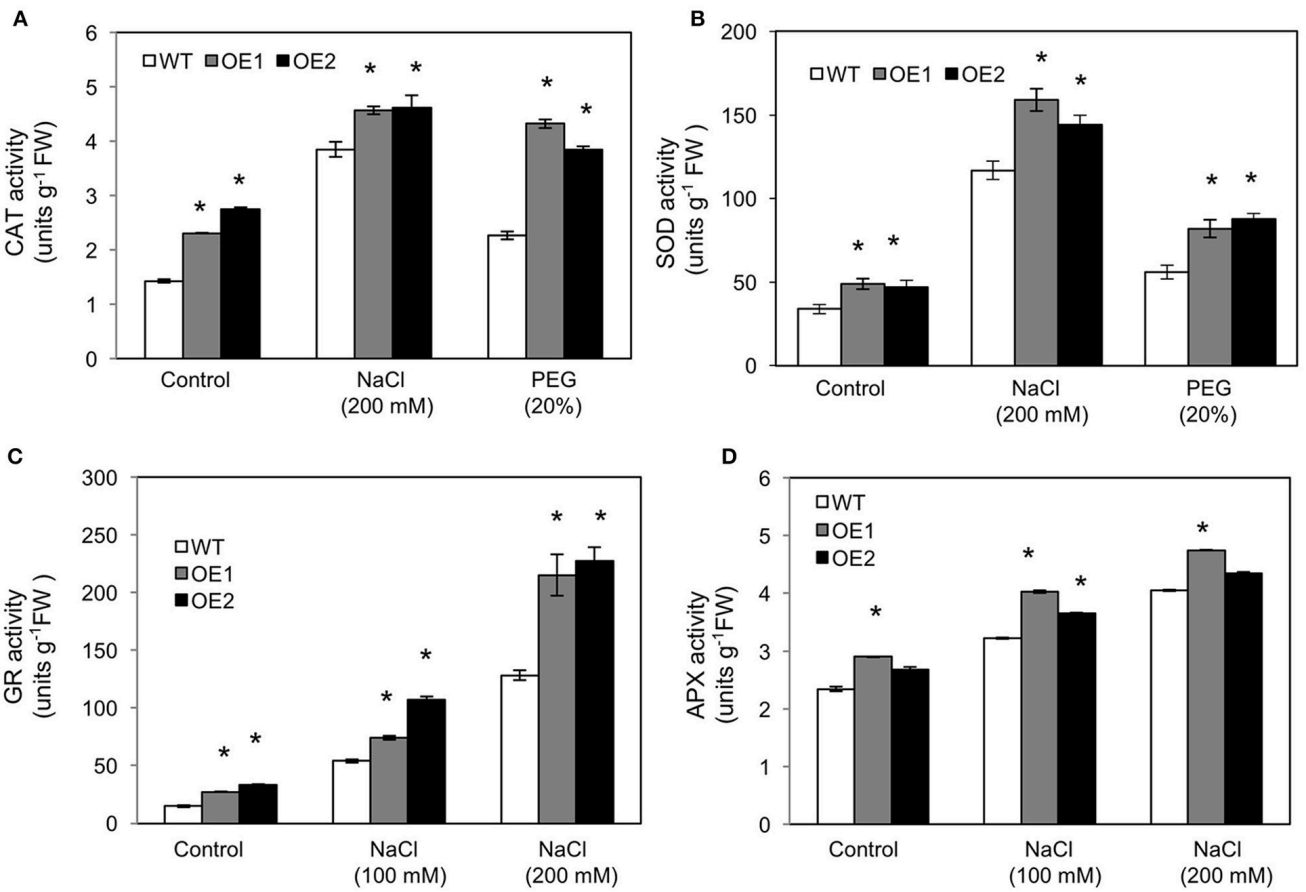
**Activities of reactive oxygen species scavenging enzymes in *HvPIP2;5* overexpressing lines under salt and osmotic stresses. (A)** Activities of catalase (CAT) in WT and OE lines after 200 mM NaCl or 20% PEG treatment. **(B)** Activities of superoxide dismutase (SOD) in WT and OE lines after 200 mM NaCl or 20% PEG treatment. **(C)** Activities of glutathione reductase after NaCl treatments in WT and OE lines. **(D)** Activities of ascorbate peroxidase after NaCl treatments in WT and OE lines. Bars indicate standard error and significant differences between WT and OE lines were marked with asterisks (*P* < 0.05).

To assess the contribution of ROS scavenging enzymes in reduction of oxidative stress caused by salt and osmotic stresses, we measured the activities of catalase (CAT) and superoxide dismutase (SOD) in WT and *HvPIP2;5* OE lines under salt and osmotic stress conditions. Although, CAT activity was elevated in both WT and *HvPIP2;5* OE lines, consistent with increase of osmotic and saline stresses (Figure 5A), the level of increase of CAT activity was significantly higher in *HvPIP2;5* OE lines than in WT. Patterns largely similar to those found in the CAT activity assay were observed in SOD activity assay. SOD activity in *HvPIP2;5* OE lines were higher than those in WT under salt and osmotic stresses (Figure 5B). Interestingly, both CAT and SOD activities in *HvPIP2;5* OE lines were generally higher than those of WT even under normal conditions.

In addition, we measured the activities of glutathione reductase (GR) and ascorbate peroxidase (APX) in *HvPIP2;5* OE lines under 100 and 200 mM NaCl conditions. GR and APX are major enzymes for the ascorbate-glutathione cycle which is an important component of the ROS scavenging system in plants (Pang and Wang, 2010). Generally, elevated activities of APX and GR have been shown to correlate with increased salt tolerance in plants (Pang and Wang, 2010). The activities of GR were higher in *HvPIP2;5* OE lines than in WT under both normal and salt conditions (Figure 5C). The activities of APX were also largely higher in *HvPIP2;5* OE lines than in WT under normal and salt conditions (Figure 5D).

Taken together, these results indicated that reduction of oxidative stress in *HvPIP2;5* OE lines under salt and osmotic stresses is possibly related to enhanced activities of ROS scavenging enzymes.

### Increased levels of proline in *HvPIP2;5* overexpressing lines

**Figure 6:**
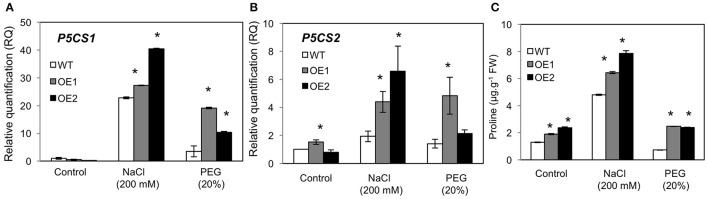
**Analysis of *P5CS1 and P5CS2* gene expression and proline levels in *HvPIP2;5* overexpressing lines under salt and osmotic stresses**. The expression levels of **(A,B)** *P5CS1* and *P5CS2* in WT and OE lines were estimated by quantitative real time PCR. Arabidopsis clatharin gene was used as an internal control for normalization. The expression levels of each gene in WT control (calibrator) were assumed as 1. Three biological replicates were averaged and bars indicate standard error. **(C)** Proline contents in WT and OE were quantified with three biological replicates. Bars indicate standard error and significant differences between WT and OE lines were marked with asterisks (*P* < 0.05).

The amino acid proline acts as an osmolytes and an antioxidant, and high levels of proline enhance stress adaptation under unfavorable conditions (Bates et al., 1973; Hayat et al., 2012). Thus, we investigated expression patterns of the Arabidopsis proline biosynthesis genes, Δ^1^-Pyrroline-5-Carboxylate Synthase 1 and 2 (*P5CS1* and *P5CS2*) in WT and *HvPIP2;5* OE lines. Real time PCR analysis revealed that gene expression levels of *P5CS1* and *P5CS2* were higher in *HvPIP2;5* OE lines than in WT under salt and osmotic stresses (Figures 6A,B). We also measured proline levels in WT and *HvPIP2;5* OE lines and found that even under normal conditions, *HvPIP2;5* OE lines demonstrated slightly higher proline levels than WT (Figure 6C). After salt treatment, proline levels were increased in both WT and *HvPIP2;5* OE lines with much higher increase in OE lines than in WT (Figure 6C). Interestingly, our PEG treatment did not seem to induce proline accumulation. Still, proline levels remained higher in the *HvPIP2;5* OE lines than in WT (Figure 6C). This salt-induced proline accumulation correlated with the up-regulation of *P5CS1* and *P5CS2* expression in *HvPIP2;5* OE lines, suggesting a possible molecular mechanism behind the enhanced stress tolerance in *HvPIP2;5* OE lines.

## Discussion

Water-deficit stress such as salt and osmotic stress and impede plant growth and development by affecting plant water balance. Aquaporins play important roles as water channels in regulating plant water status; thus, many aquaporin genes from diverse plant species have been used in transgenic research to improve water-deficit stress tolerance in plants (Martre et al., 2002; Cui et al., 2008; Khan et al., 2015).

We found that overexpression of barley *PIP2;5* (*HvPIP2;5*) in both yeast and Arabidopsis improved tolerance to high salt and high osmotic stresses (Figures 1–3). These improved tolerances in *HvPIP2;5* overexpressing Arabidopsis were also observed in milder stress conditions (100 mM NaCl and 10% PEG) (Supplementary Figures 3, 4). Increased stress tolerance in *HvPIP2;5* overexpressing Arabidopsis was correlated with lower levels of stress-induced ROS (Figure 4 and Supplementary Figure 5), high activity of ROS scavenging enzymes (Figure 5), higher induction of proline biosynthetic gene expression and high levels of osmoprotectant proline (Figure 6). In particular, *HvPIP2;5* overexpressing Arabidopsis displayed lower water loss in shoots and higher relative water contents in roots than did WT under salt and osmotic stresses.

While *HvPIP2;5* overexpression improved water-stress tolerance in both yeast and Arabidopsis, Arabidopsis *PIP2;5* (*AtPIP2;5*) overexpression brought about reduced osmotic stress tolerance in Arabidopsis and tobacco (Jang et al., 2007). These seemingly contradicting results are not uncommon. In fact, many aquaporin overexpression studies have produced contrasting results—aquaporin-overexpressing plants have shown either positive or negative effects on stress tolerance (Maurel et al., 2008; Martinez-Ballesta and Carvajal, 2014; Zhou et al., 2014). Even overexpression of aquaporins with high homology has resulted in different sensitivities to dehydration stress. For example, Arabidopsis *AtPIP1;2*, rice *RWC3*, and tobacco *NtAQP1* share approximately 80% sequence identity. Despite this, overexpression of *AtPIP1;2* in tobacco has caused reduced stress tolerance (Aharon et al., 2003) while *RWC3* overexpression in rice and *NtAQP1* overexpression in tomato have shown enhanced tolerance under drought and salt stress, respectively (Lian et al., 2004; Sade et al., 2010). In addition, Arabidopsis *pip2;2* mutants display defects in hydraulic conductivity despite the expression of a very close homolog AtPIP2;3 which shares >96% homology, demonstrating that close aquaporin homologs could not function redundantly even within the same plant (Javot et al., 2003).

Although, *HvPIP2;5* and *AtPIP2;5* share high sequence homology, there are differences in gene regulation. *AtPIP2;5* expression levels remain low in Arabidopsis and are only up-regulated by drought and cold (Jang et al., 2007). However, *HvPIP2;5* is one of the highly expressed *PIP*s in barley which is down-regulated by osmotic stress (Katsuhara et al., 2014). These different patterns of gene expression might indicate the divergent functions of *PIP2;5* in Arabidopsis and barley, and may be attributed to contrasting stress responses in *HvPIP2;5* and *AtPIP2;5* overexpressors.

Another explanation for the contrasting results might lie in the difference in protein sequence between HvPIP2;5 and AtPIP2;5 proteins. Amino acid sequence differences are mainly found in the N-terminus which is expected to be exposed on the cytosol side (Walz et al., 1997; Supplementary Figures 1A, 6). Thus, it is tempting to speculate that contrasting stress phenotypes may be due to differences in the N-termini of PIP2;5 proteins which may contain important motifs such as for activity regulation, protein stability, protein interaction, or even subcellular localization. Although transcriptional control of aquaporins appeared to be important for physiological functions (Alexandersson et al., 2005; Guo et al., 2006; Jiang et al., 2016), aquaporin activity is also post-translationally regulated by protein modification including phosphorylation (Johansson et al., 1998; Santoni et al., 2003; Daniels and Yeager, 2005). We have found some differences in phosphorylation sites at the N-terminus of two PIP2;5 proteins (Supplementary Figure 6) which might be important for PIP2;5 function. This differential protein modification might cause variable functional activities of PIP2;5 in plant tissue, particularly where proteins are ectopically expressed due to constant promoter activity. It is interesting to note that Arabidopsis PIP2;1, an AtPIP2;5 close homolog was shown to be a drought response-negative regulator which is a target of ubiquitination and degradation by a RING membrane-anchor 1 E3 ubiquitin ligase (Lee et al., 2009). Thus, it might be possible that this kind of Arabidopsis regulation system might function differently on the endogenous PIP2;5 (AtPIP2;5) and the heterologous PIP2;5 (HvPIP2;5) due to the different amino acid residues in the N-termini.

We found, in *HvPIP2;5* overexpressing Arabidopsis, up-regulation of *P5CS2*, high levels of proline, and increased activities of SOD, CAT, GR, and APX with reduced levels of ROS under drought and salt stress conditions. Particularly, up-regulation of the key proline biosynthetic *P5CS* genes coincided with increased levels of osmoprotectant proline under salt stress (Figure 6). Proline mainly functions in defense and turgor pressure maintenance against water-deprived conditions (Oregan et al., 1993; Kishor et al., 1995; Khedr et al., 2003). Thus, enhanced stress tolerance in *HvPIP2;5* overexpressing plants seemed to result, at least in part, from increased expression of proline biosynthetic genes and elevated activities of ROS scavenging enzymes. Similar to our findings, the overexpression of wheat *TaAQP7*, one of the closest homologs of *HvPIP2;5*, in tobacco enhanced drought tolerance in correlation with decreased levels of MDA and H_2_O_2_ and increased activities of SOD and CAT enzymes (Zhou et al., 2012). Improved osmotic stress tolerance by up-regulation of stress-induced genes and an increase of ROS scavenging enzyme activity suggest that *HvPIP2;5* overexpression might sensitize transgenic plants, making the overexpressors react faster to osmotic stress signals and eventually induce enhanced stress defense. Thus, one might speculate that *HvPIP2;5* aquaporins in transgenic plants might activate osmotic and salt stress sensing or upstream steps in signaling pathways to induce better stress-tolerance mechanism than WT under stress conditions. Consistent with our speculation, it has been suggested that aquaporins may be part of an osmotic stress signaling cascade (Maurel et al., 2008); additionally, it has even been proposed that aquaporins may act as osmosensors (Hill et al., 2004). Further, study will be required to investigate this possibility.

In conclusion, we have shown that *HvPIP2;5* can improve tolerance to salt and osmotic stresses when overexpressed in yeast and Arabidopsis. Our results contrast with a previous *AtPIP2;5* overexpression study where osmotic stress sensitive phenotypes in *AtPIP2;5* overexpressing plants were reported (Jang et al., 2007). These results suggest the diversity of PIP regulation and function in acquiring stress tolerance in plants. Further studies should be conducted to understand the functional differences among aquaporins for crop improvement under abiotic stress.

## Author contributions

HA, BhL, and SKP designed the experiments; HA, JPA performed the experiments; GR, LS, BhL, and SKP advised the research; HA, BhL, and SKP discussed the results and wrote the paper.

### Conflict of interest statement

The authors declare that the research was conducted in the absence of any commercial or financial relationships that could be construed as a potential conflict of interest.

## Supplementary material

Supplementary Table 1 **Sequences of primers used in the study**.

Supplementary Figure 1 **Sequence analysis and structure prediction of HvPIP2;5 protein. (A)** Alignment of full-length deduced amino acid sequences of HvPIP2;5 and its Arabidopsis homolog, AtPIP2;5. Asterisks below the alignment indicate identical amino acids. The MIPS domain is marked with a black bar below the alignment. The highly conserved amino acid sequence “HINPAVTFG” and two NPA motifs are marked with a solid-line box and a dotted-line box, respectively. The conserved phosphorylation-target serine residues in the C-termini were shown in bold. **(B)** Six predicted transmembrane helical regions (H1–H6) were indicated with boxes in the protein sequence alignments in HvPIP2;5 and AtPIP2;5 sequences. The prediction was made by TMPred online software. **(C)** HvPIP2;5 secondary structure prediction using SOPMA online program. **(D)** Phylogenetic analysis of members of barley PIP2 clade aquaporins. GenBank accession numbers for barley PIP2 clade proteins are as follows: HvPIP2;1 (BAE02729.1), HvPIP2;2 (BAG06230.1), HvPIP2;3 (BAF33069.1), HvPIP2;4 (BAE06148.1), HvPIP2;5 (BAG06231.1), HvPIP2;7 (ADW85675.1), HvPIP2;8 (BAJ90410.1), HvPIP2;9 (BAJ92749.1), HvPIP2;10 (BAK04917.1). Neighbor-end joining (NJ) method was applied using MEGA6 with a tree file produced by Clustal omega. The scale bar indicates 0.2 substitutions per site.

Supplementary Figure 2 **Generation of *HvPIP2;5* overexpressing Arabidopsis. (A)** Schematic illustration of the binary vector region used for *HvPIP2;5* overexpression in Arabidopsis. The complete ORF of *HvPIP2;5* was cloned in pCAMBIA-2301 vector carrying CaMV35s promoter. **(B)** Detection of the *HvPIP2:5* transcripts from the *HvPIP2;5* overexpressing Arabidopsis lines by semi-quantitative RT-PCR. Ubiquitin gene was used as an internal control.

Supplementary Figure 3 **Seed germination and root growth of *HvPIP2;5* overexpressing lines. (A,C)** Seed germination of WT and OE lines on MS media containing 100 mM NaCl or 10% PEG. **(B,D)**, Root elongation on WT and OE lines on MS media containing 100 mM NaCl or 10% PEG.

Supplementary Figure 4 **Chllorophyll and relative water contents in *HvPIP2;5* overexpressing lines. (A)** Chlorophyll A contents under salt (100 mM) or osmotic stress (10% PEG) conditions. **(B)** Relative water contents under salt (100 mM) or osmotic stress (10% PEG) conditions. Bars represent means ± SE and values with asterisks indicate significance at *P* < 0.05.

Supplementary Figure 5 **Analysis of superoxide, hydrogen peroxide, malondialdehyde contents in *HvPIP2;5* overexpressing lines under salt and osmotic stresses. (A)** Quantification of superoxide contents in WT and OE lines after 100 mM NaCl or 10% PEG treatments. **(B)** Quantification of hydrogen peroxide contents in WT and OE lines after 100 mM NaCl or 10% PEG treatments. **(C)** Quantification of MDA (malondialdehyde) levels in WT and OE lines after 100 mM NaCl or 10% PEG treatments. Bars indicate standard error and significant differences between WT and OE lines were marked with asterisks (*P* < 0.05).

Supplementary Figure 6 **Predicted phosphorylation sites in PIP2;5 proteins**. Predicted phosphorylation sites in **(A)** HvPIP2;5 and **(B)** AtPIP2;5 (At3554820). Phosphorylation site prediction was carried out using NetPhos 2.0 (http://www.cbs.dtu.dk/services/NetPhos/). Peaks above the threshold line (red) indicate the phosphorylation sites with high probability.
