# Supplementary figures and images for: Overexpression of a Barley Aquaporin Gene, HvPIP2;5 Confers Salt and Osmotic Stress Tolerance in Yeast and Plants
Source: Front Plant Sci. 2016 Oct 21;7:1566. doi: 10.3389/fpls.2016.01566 (PMC5073208; doi:10.3389/fpls.2016.01566)

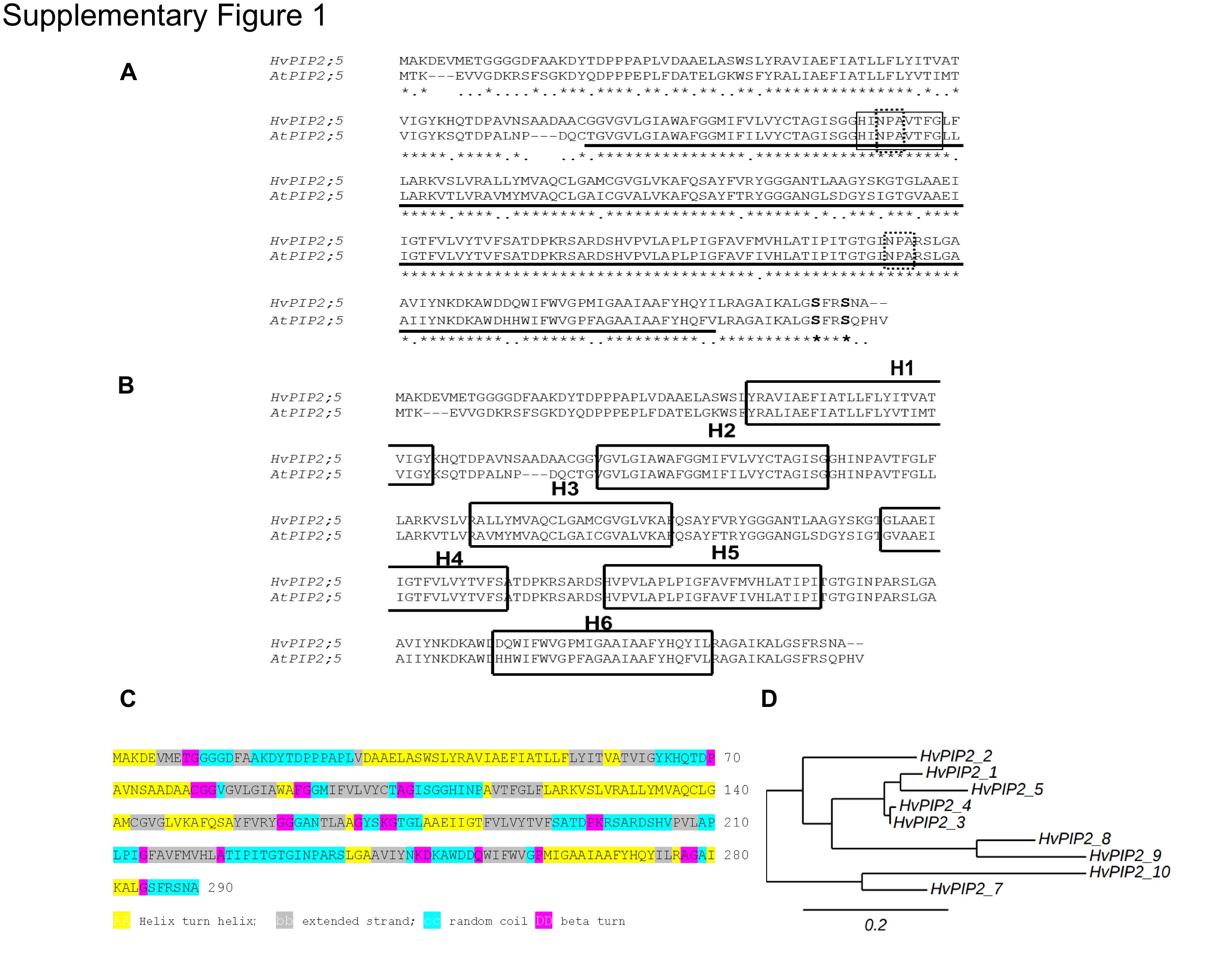

Supplement: Supplementary Figure 1 — Sequence analysis and structure prediction of HvPIP2;5 protein. (A) Alignment of full-length deduced amino acid sequences of HvPIP2;5 and its Arabidopsis homolog, AtPIP2;5. Asterisks below the alignment indicate identical amino acids. The MIPS domain is marked with a black bar below the alignment. The highly conserved amino acid sequence “HINPAVTFG” and two NPA motifs are marked with a solid-line box and a dotted-line box, respectively. The conserved phosphorylation-target serine residues in the C-termini were shown in bold. (B) Six predicted transmembrane helical regions (H1–H6) were indicated with boxes in the protein sequence alignments in HvPIP2;5 and AtPIP2;5 sequences. The prediction was made by TMPred online software. (C) HvPIP2;5 secondary structure prediction using SOPMA online program. (D) Phylogenetic analysis of members of barley PIP2 clade aquaporins. GenBank accession numbers for barley PIP2 clade proteins are as follows: HvPIP2;1 (BAE02729.1), HvPIP2;2 (BAG06230.1), HvPIP2;3 (BAF33069.1), HvPIP2;4 (BAE06148.1), HvPIP2;5 (BAG06231.1), HvPIP2;7 (ADW85675.1), HvPIP2;8 (BAJ90410.1), HvPIP2;9 (BAJ92749.1), HvPIP2;10 (BAK04917.1). Neighbor-end joining (NJ) method was applied using MEGA6 with a tree file produced by Clustal omega. The scale bar indicates 0.2 substitutions per site. [file Image1.JPEG]

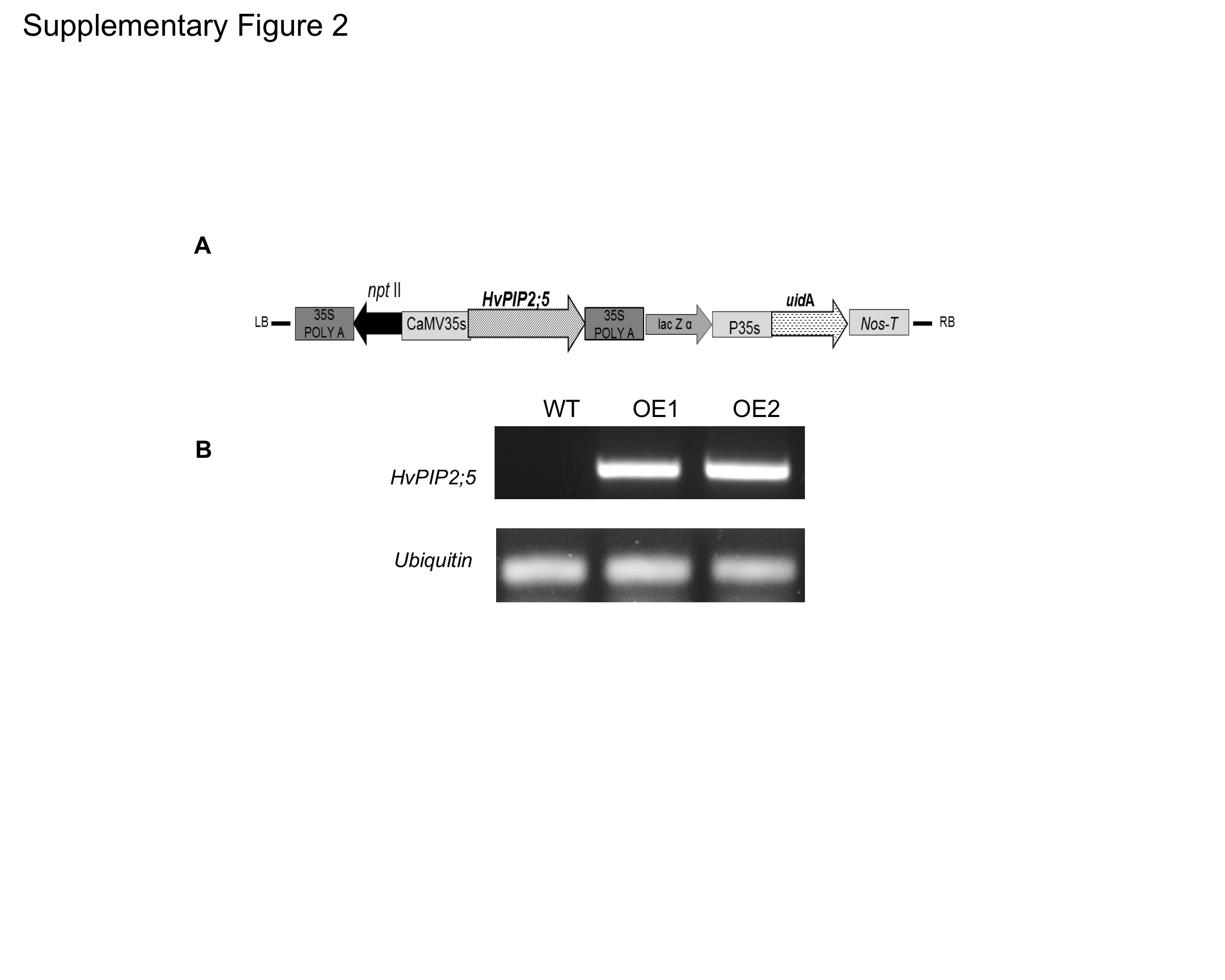

Supplement: Supplementary Figure 2 — Generation of HvPIP2;5 overexpressing Arabidopsis. (A) Schematic illustration of the binary vector region used for HvPIP2;5 overexpression in Arabidopsis. The complete ORF of HvPIP2;5 was cloned in pCAMBIA-2301 vector carrying CaMV35s promoter. (B) Detection of the HvPIP2:5 transcripts from the HvPIP2;5 overexpressing Arabidopsis lines by semi-quantitative RT-PCR. Ubiquitin gene was used as an internal control. [file Image2.JPEG]

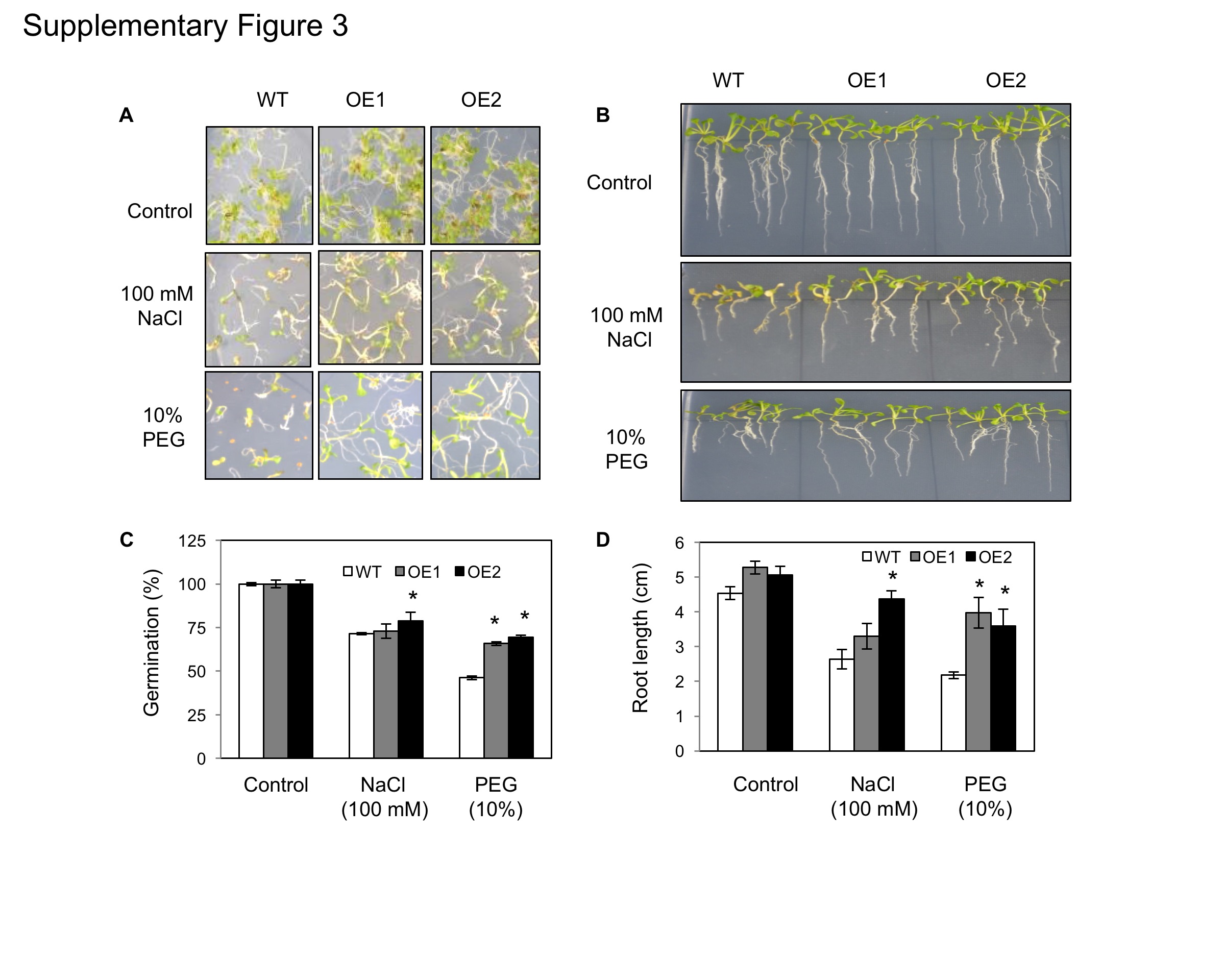

Supplement: Supplementary Figure 3 — Seed germination and root growth of HvPIP2;5 overexpressing lines. (A,C) Seed germination of WT and OE lines on MS media containing 100 mM NaCl or 10% PEG. (B,D), Root elongation on WT and OE lines on MS media containing 100 mM NaCl or 10% PEG. [file Image3.JPEG]

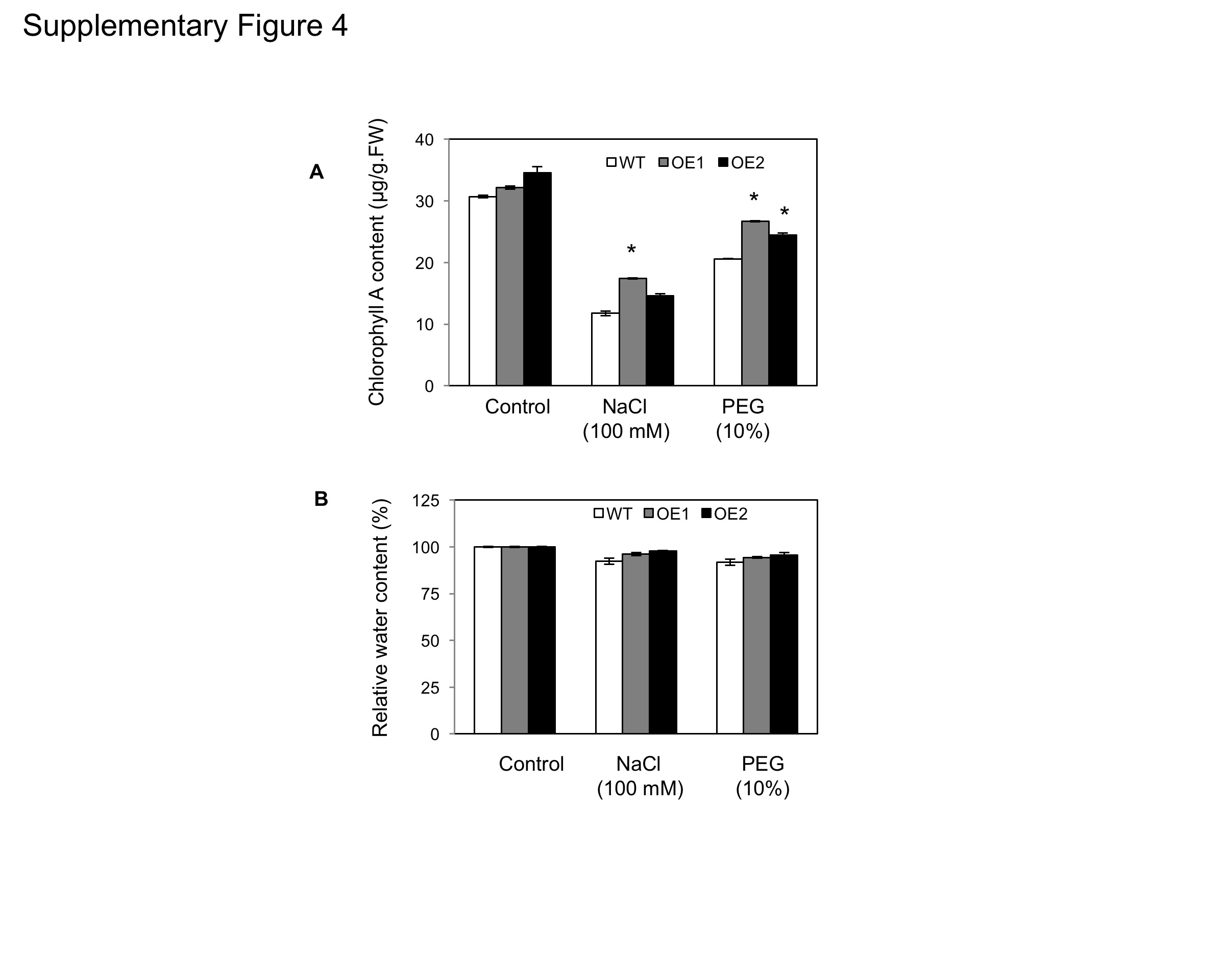

Supplement: Supplementary Figure 4 — Chllorophyll and relative water contents in HvPIP2;5 overexpressing lines. (A) Chlorophyll A contents under salt (100 mM) or osmotic stress (10% PEG) conditions. (B) Relative water contents under salt (100 mM) or osmotic stress (10% PEG) conditions. Bars represent means ± SE and values with asterisks indicate significance at P < 0.05. [file Image4.JPEG]

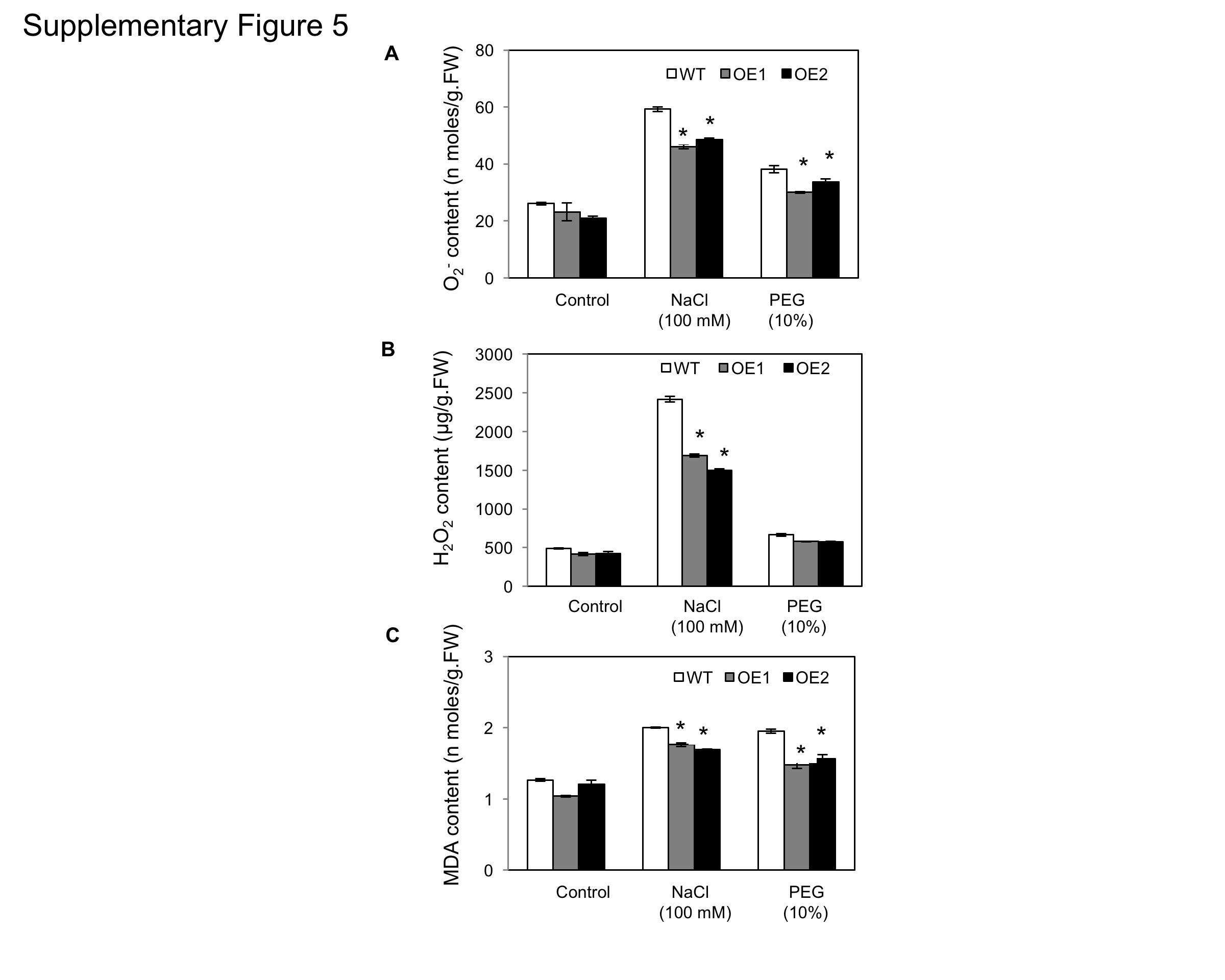

Supplement: Supplementary Figure 5 — Analysis of superoxide, hydrogen peroxide, malondialdehyde contents in HvPIP2;5 overexpressing lines under salt and osmotic stresses. (A) Quantification of superoxide contents in WT and OE lines after 100 mM NaCl or 10% PEG treatments. (B) Quantification of hydrogen peroxide contents in WT and OE lines after 100 mM NaCl or 10% PEG treatments. (C) Quantification of MDA (malondialdehyde) levels in WT and OE lines after 100 mM NaCl or 10% PEG treatments. Bars indicate standard error and significant differences between WT and OE lines were marked with asterisks (P < 0.05). [file Image5.JPEG]

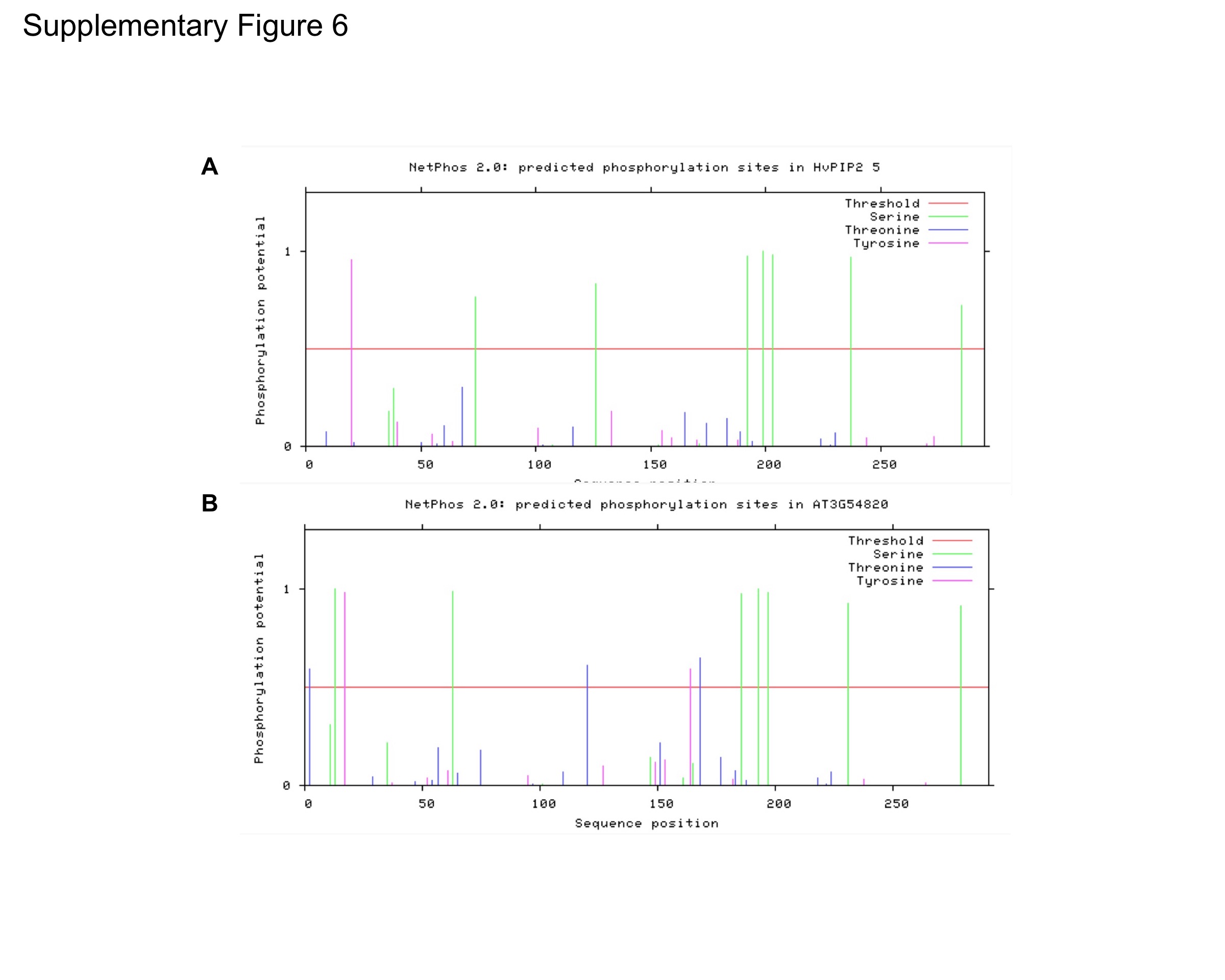

Supplement: Supplementary Figure 6 — Predicted phosphorylation sites in PIP2;5 proteins. Predicted phosphorylation sites in (A) HvPIP2;5 and (B) AtPIP2;5 (At3554820). Phosphorylation site prediction was carried out using NetPhos 2.0 (http://www.cbs.dtu.dk/services/NetPhos/). Peaks above the threshold line (red) indicate the phosphorylation sites with high probability. [file Image6.JPEG]
